# Clinical, ultrasonographic, and postmortem findings in sheep and goats with urinary tract disorders

**DOI:** 10.14202/vetworld.2021.1879-1887

**Published:** 2021-07-20

**Authors:** Mohamed Tharwat

**Affiliations:** 1 Department of Veterinary Medicine, College of Agriculture and Veterinary Medicine, Qassim University, P.O. Box 6622, Buraidah, 51452, Saudi Arabia; 2 Department of Animal Medicine, Faculty of Veterinary Medicine, Zagazig University, 44519, Zagazig, Egypt

**Keywords:** goat, pathology, sheep, ultrasonography, urinary

## Abstract

**Background and Aim:**

In sheep and goats, most urinary disorders are incidental findings at the postmortem examination and do not present clinically. As such, the diagnosis of renal diseases in sheep and goats can be overlooked. Therefore, this study was carried out on sheep and goats to describe the clinical, ultrasonography(USG), and postmortem findings of various disorders affecting the urinary tract.

**Materials and Methods:**

A total of 58 animals (30 sheep and 28 goats) were examined, as well as were ten healthy control animals (five sheep and five goats) for a comparison. The animals were referred for various causes, including anorexia, frequent and painful urination, hematuria, decreased body weight, oliguria, and anuria.

**Results:**

The sheep and goats were categorized into ten groups of symptoms and disorders: Pelvic abscessation, pigmented urine, renal failure, pyelonephritis, cystitis, hydronephrosis, rupture of the urethra, rupture of the urinary bladder, rupture of the urinary bladder together with the urethra, and paralysis of the urinary bladder. Clinical presentation, USG, and postmortem examination results for each group were detailed. Many clinical presentations were nonspecific. USG examination of the urinary tract significantly facilitated the verification of the previously mentioned disorders.

**Conclusion:**

USG demonstrated superior performance in the early and confirmatory diagnosis of urinary disorders in sheep and goats. Therefore, it is recommended that routine USG be the preferred imaging modality for examining sheep and goats with urinary disorders and for determining their prognosis.

## Introduction

Ultrasonography (USG) is commonly used in large animals to determine the diagnosis of and prognosis for disorders in various regions, including thoracic [1,2] hepatic [3], and abdominal [4,5]. Diagnostic USG also has been used in large animals to diagnose various renal disorders [6-9]. In the majority of reported cases, a confirmatory diagnosis would have been difficult to reach without USG.

In small ruminants, USG is commonly used at the farm level for gynecological and obstetrical purposes [10]. USG also has been used to diagnose thoracic [11], hepatic [12], and abdominal [13] afflictions. In sheep, USG findings for obstructive urolithiasis in rams have been reported [14,15]. Scott [16] has presented transabdominal USG images of the bladder and right kidney of sheep with suspected urinary tract disease, concluding that this imaging can assist in treatment decisions.

However, reports describing USG findings in goats with urinary afflictions are rare. Only two female goats with cystitis, pyelonephritis, and obstructive uropathy have been described in the literature [17,18]. Modern portable USG devices provide veterinary practitioners an inexpensive and non-invasive method to further examine sheep and goats on farms. The process takes no more than a few minutes, with immediate results. Repeat examinations allow the monitoring of disease progression and the assessment of therapy [19].

Early diagnosis of renal afflictions in sheep and goats would be useful for early medical or surgical intervention, improving animal prognosis and welfare. Therefore, this study describes the clinical, USG, and postmortem findings of various urinary tract disorders in goats and sheep.

## Materials and Methods

### Ethical approval

The Animal Ethical Committee of the Deanship for Scientific Research, Qassim University, Saudi Arabia approved this study design. Animals were maintained and treated according to the Laboratory Animal Control Guidelines of Qassim University, which basically conforms to the Guide for the Care and Use of Laboratory Animals of the National Institutes of Health in the USA (NIH publications No. 86 to 23, revised 1996).

### Study period and location

The study was conducted from October 2008 to March 2019 at Qassim University Veterinary Teaching Hospital in Saudi Arabia.

### Animals

A total of 58 animals (30 sheep and 28 goats) were examined at Qassim University Veterinary Teaching Hospital in Saudi Arabia. The animals were referred for various causes, including anorexia, frequent and painful urination, red urine, decreased body weight, oliguria, and anuria. Ten healthy animals (five sheep and five goats) were examined and used as a control group. All animals underwent a thorough physical examination, which included general behavior and condition; auscultation of the heart, lungs, rumen, and intestines; measurement of heart and respiratory rates; rectal temperature and examination; swinging auscultation; and percussion auscultation of both sides of the abdomen.

### USG and postmortem examination

The 10^th^-12^th^ intercostal spaces on the animals’ right and both flanks were clipped, and the skin was shaved. Transcutaneous USG of the kidneys and the urinary bladder was done using a real-time scanner (SSD-500, Aloka, Tokyo, Japan) equipped with 3.5- and 7.5-MHz sector transducers. The examination was conducted on standing, non-sedated animals, as described previously [14]. The kidneys were examined in longitudinal and cross-section and were assessed subjectively. For the right kidney, the last two intercostal spaces on the right and the region immediately caudal to the last rib were scanned. The right and left dorsal flanks were scanned to locate the left kidney. The bladder was evaluated transcutaneously by scanning the right and left inguinal regions with the animal in the recumbent position. Following the USG examination, a thorough postmortem examination of the urinary tract in the sick animals was performed.

## Results

None of the control sheep and goats had urinary tract abnormalities detected by either clinical or USG examinations. The diseased animals (n=58; 30 sheep and 28 goats) were categorized into ten groups according to final diagnosis (Table-1).

**Table-1:** Classification of sheep and goats with different urinary disorders.

Group	Category	Total number	Sheep	Goats
1	Pelvic abscesses	3	2 (ram and ewe)	1 (goat)
2	Red urine	7	2 (rams)	5 (bucks)
3	Renal impairment	3	1 (ewe)	2 (buck and goat)
4	Pyelonephritis	4	2 (rams)	2 (bucks)
5	Cystitis	9	5 (3 ewes, 2 rams)	4 (3 bucks, 1 goat)
6	Hydronephrosis	9	6 (4 rams, 2 ewes)	3 (bucks)
7	Rupture of the urethra	12	7 (rams)	5 (bucks)
8	Rupture of the urinary bladder	6	2 (ram, female lamb)	4 (bucks)
9	Rupture of the urinary bladder together with urethra	3	2 (rams)	1 (buck)
10	Paralysis of the urinary bladder	2	1 (male lamb)	1 (goat)
Total		58	30	28

In Group-1, the animals with pelvic abscesses presented with a history of body weight loss. Clinical examination revealed pale mucus membranes, tachycardia, polypnea, and stranguria. Abdominal scanning revealed a distended urinary bladder containing echogenic deposits and a dilated renal pelvis and calices. The pelvic abscesses in the goats and ram of this group were close to the bladder. The content of the lesions was hyperechoic, and the bladder contained ventrally located hyperechoic sludge. Abscess formation was confirmed through pus aspiration. The bladder in the ram contained deep-red urine. Postmortem findings in one sheep included an abscess, distension containing deep-red urine, clotted blood, and ulcerated mucosa in the bladder; a dilated renal pelvis; and hydropericardium.

**Figure-1:**
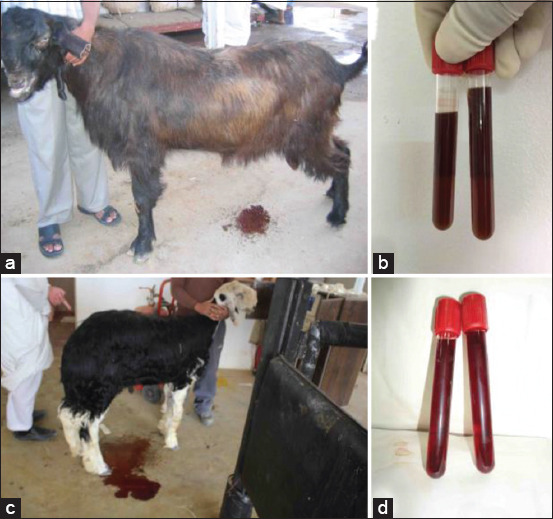
Red urine in a buck (a) and a ram (c) following grazing in a pasture contained toxic plants. Images (b) and (d) show urine after centrifugation. No sediments were formed, confirming hemoglobinuria.

In Group-2, the animals with red urine presented with a history of body weight loss and discolored urine confirmed as hemoglobinuria by centrifugation (no sedimentation) (Figure-1). The owner reported that poisonous plants had been detected in the grazing area.

**Figure-2:**
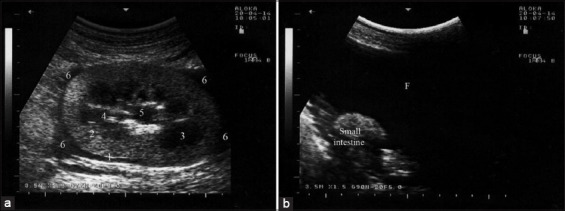
Ultrasonographic findings in a buck with renal impairment using a 3.5 MHz convex probe: (a) Increased echogenicity of the renal cortex; (b) peritoneal effusion of anechoic fluid (F). (1, renal capsule; 2, renal cortex; 3, medullary pyramids; 4, inter-lobar vessels; 5, renal sinus; and 6, peri-renal fluid).

The animals in Group-3 presented with a history of dullness, depression, and gradual abdominal distension over the previous week. A complete blood count showed neutrophilic leukocytosis. Blood chemistry revealed remarkable increases in BUN (blood urea nitrogen) (64.3 mmol/L; reference range, 3.0-10.0 mmol/L) and creatinine (1637 mmol/L; reference range, 70-105 mmol/L). Abdominocentesis of the lower abdomen revealed a clear serous fluid. USG of the abdomen showed viscera floating within the anechoic fluid. Transcutaneous USG of the right kidney showed increased echogenicity of the renal cortex (Figure-2). Unfortunately, a postmortem examination was not done with this group because the animals were discharged from the hospital at the owners’ request.

**Figure-3:**
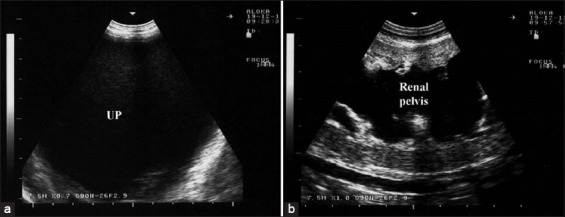
Ultrasonographic findings in a male goat with pyelonephritis using a 7.5 MHz convex probe: (a) Uroperitoneum (UP); (b) markedly increased echogenicity of the renal parenchyma with dilated renal pelvis.

**Figure-4:**
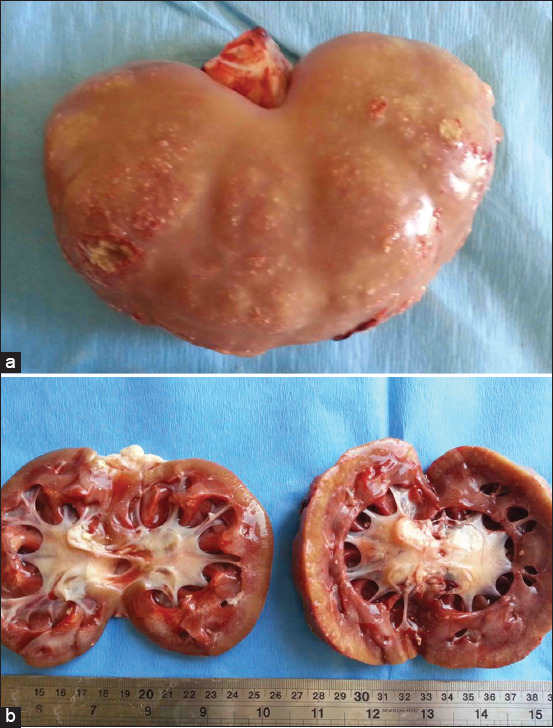
Postmortem finding of a buck with pyelonephritis; (a) sub-capsular pinpoint microabscesses; (b) bilateral dilatation of the renal pelvises.

In Group-4, two bucks and two rams with pyelonephritis presented with a history of dullness, depression, abdominal distension, and dysuria. USG of the abdomen revealed echogenic fluid. Transcutaneous USG of both kidneys showed a markedly increased echogenicity of the renal parenchyma, with fluctuating debris in the dilated cystic renal pelvis and calices (Figure-3). Postmortem findings in one goat included a massive amount of red urine within the abdominal cavity, renal bilateral, sub-capsular pinpoint microabscesses, and bilateral hydronephrosis (Figure-4). Pyelonephritis was confirmed histologically.

**Figure-5:**
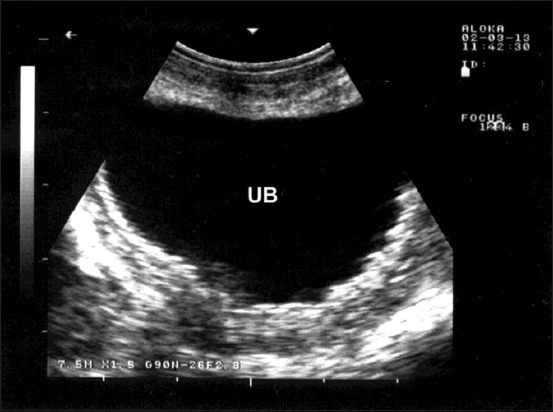
Ultrasonographic findings in a goat with cystitis showing the thickened and corrugated mucosa of the urinary bladder (UB). Image was taking using a 7.5 MHz convex transducer.

In Group-5, the sheep and goats with urinary bladder inflammation presented with frequent painful urination and a prolonged urination posture. USG showed thickened and corrugated bladder mucosa (Figure-5).

**Figure-6:**
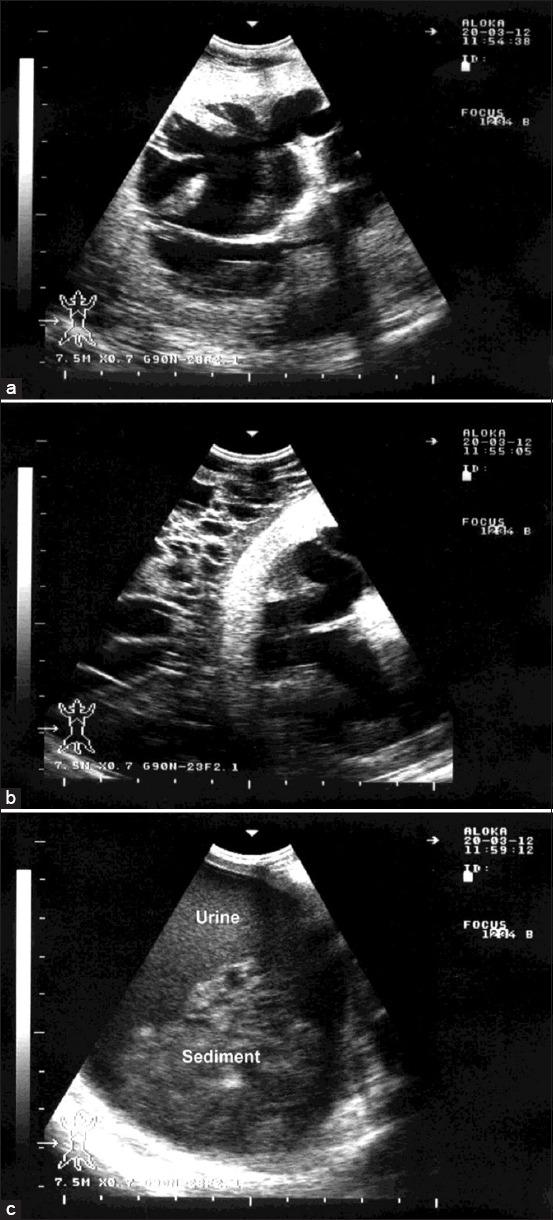
Ultrasonographic findings in a ram with hydronephrosis using a 7.5 MHz convex transducer: (a) Severely enlarged kidney with dilated renal pelvis and pressure atrophy of the renal parenchyma; (b) perirenal edema; (c) ventral sludge in the urinary bladder.

**Figure-7:**
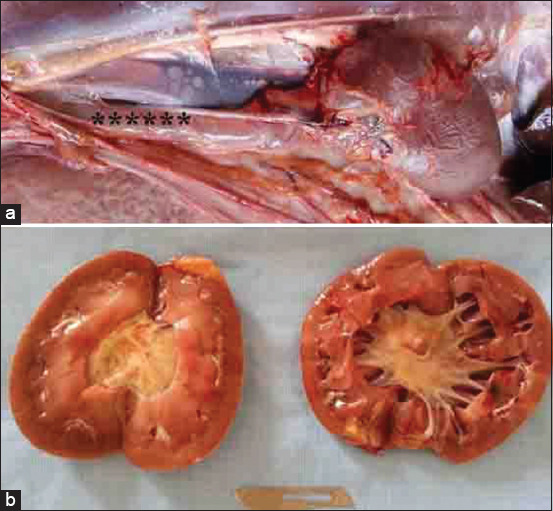
Postmortem findings of a sheep with unilateral hydronephrosis: (a) Notable dilated right ureter (black stars) and its corresponding kidney; (b) unilateral dilatation of the renal pelvis compared to a relatively normal left kidney.

In Group-6, the animals with hydronephrosis presented with loss of body condition. USG examination revealed dilated renal pelvises, pressure atrophy of the renal parenchyma, perirenal edema, and ventral sludge in the urinary bladder (Figure-6). With the exception of unilateral hydronephrosis in one ewe (Figure-7), the lesions in the remaining animals were bilateral.

**Figure-8:**
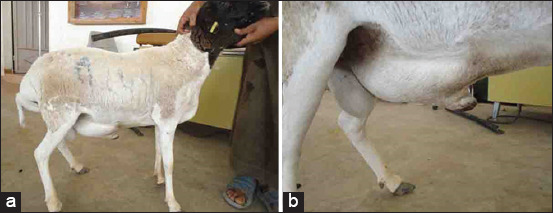
(a) Ruptured urethra in a ram; (b) close-up view of the enlarged penile body and testicles.

**Figure-9:**
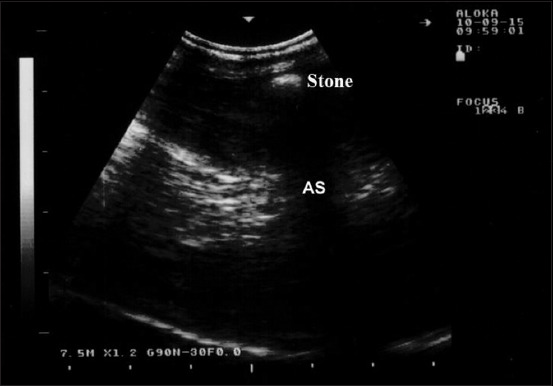
Ultrasonographic findings in a ram with ruptured urethra; a calculus within the penile body appears as an acoustic enhancement (stone) with distal acoustic shadowing (AS). Image was taking using a 7.5 MHz convex transducer.

**Figure-10:**
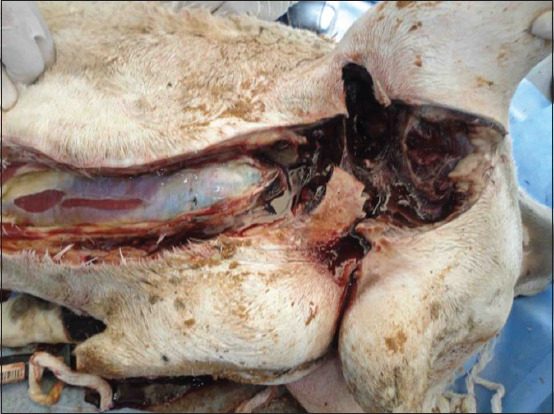
Postmortem finding of the ram with ruptured urethra in Figure-12 showing muscle necrosis and gangrene and subcutaneous urine accumulation.

**Figure-11:**
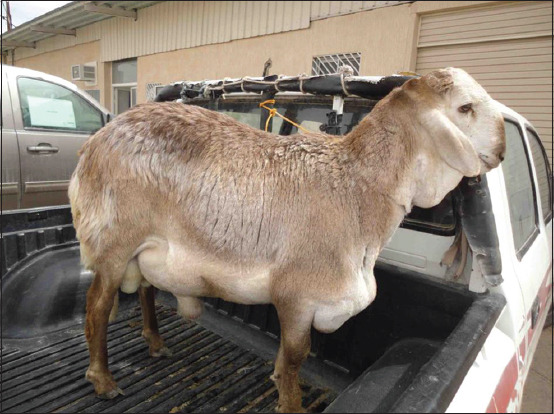
Ruptured urethra in a ram; enlarged penile body and testicles are evident.

**Figure-12:**
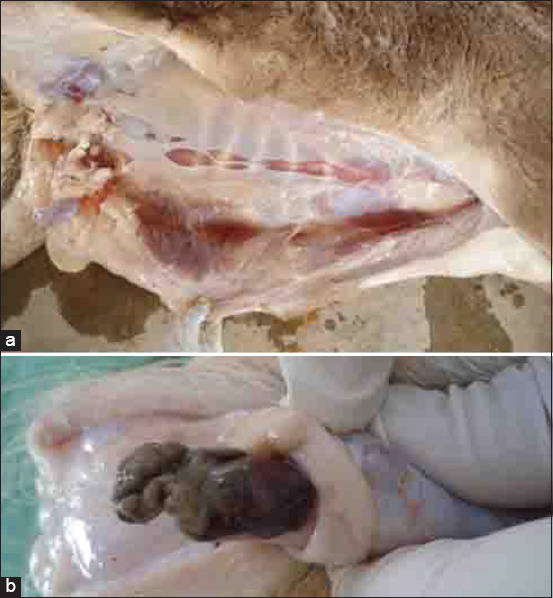
Postmortem findings of the ram with ruptured urethra in Figure-14: (a) urine accumulated within the muscles leading to gangrene of the muscles; (b) gangrene of the penile body.

The rams and bucks in Group-7 with ruptured urethras presented with anorexia, dullness, depression, and anuria. Clinical examination revealed an enlarged and edematous penis and testicles (Figure-8), with an offensive uremic odor emanating from the mouth. USG revealed anechoic fluid within the testes and fluid-filled pockets indicating urine in the subcutaneous tissue. USG in another case showed a calculus within the penile body that appeared as an acoustic enhancement with distal acoustic shadowing (Figure-9). Based on the clinical and USG findings, a ruptured urethra was diagnosed, along with a grave prognosis. Postmortem findings in the ram included gangrene of both the muscles where the urine had accumulated and of the penile body (Figure-10). Clinical examination of another ram with a ruptured urethra revealed an enlarged and edematous penis and testicles, with an offensive uremic odor emanating from the mouth. Urine also had accumulated subcutaneously around the penis, ventral abdomen, and anterior to the thorax (Figure-11). Postmortem findings in this ram included accumulation of reddish urine ventral to the abdomen. The urethral process and glans penis were necrotic (Figure-12).

**Figure-13:**
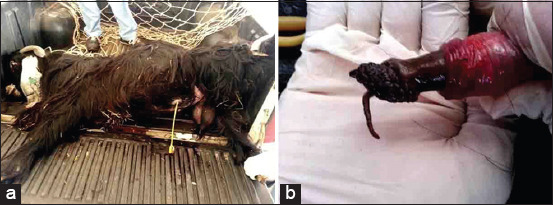
Ruptured urinary bladder in an adult buck: (a) Reddish urine revealed by abdominocentesis that yielded red sediment after centrifugation; (b) congested and cyanosed urethral process and glans penis.

**Figure-14:**
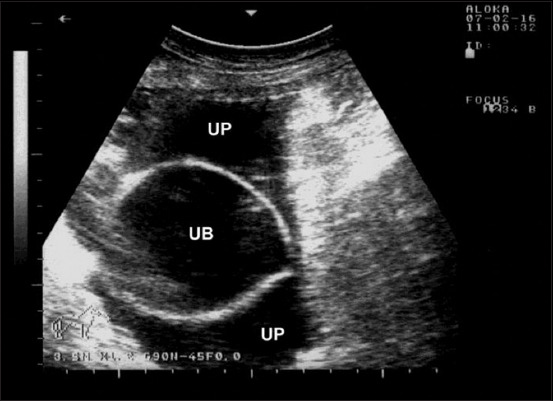
Ultrasonography of the buck with ruptured urinary bladder in Figure-13 showing a collapsed urinary bladder (UB) with uroperitoneum (UP). Image was taking using a 3.5 MHz convex transducer.

In Group-8, animals with a ruptured urinary bladder presented with anorexia, dullness, depression, abdominal distension, and anuria, with an offensive uremic odor emanating from the mouth. A congested and cyanosed urethral process and glans penis were found in one buck (Figure-13). Abdominocentesis revealed reddish urine, with erythrocyte sedimentation forming after centrifugation. Abdominal USG showed a collapsed bladder floating in anechoic fluid (uroperitoneum) (Figure-14). Postmortem examination of a 2-month-old female lamb included uroperitoneum, pelvic abscessation obstructing the bladder neck, and bilateral hydronephrosis.

**Figure-15:**
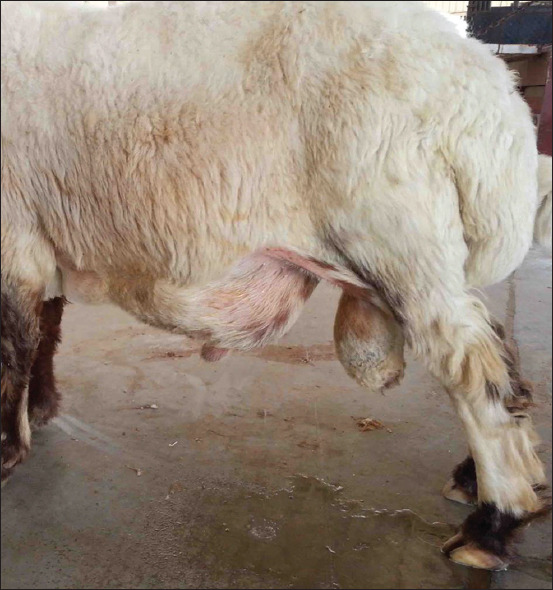
Ruptured urinary bladder and ruptured urethra in a ram. Note: Abdominocentesis yielded urine.

In Group-9, the rams and buck with ruptured urethras and urinary bladders presented with anorexia, dullness, depression, and anuria. Clinical examination revealed an enlarged and edematous penis and testicles. Urine also had accumulated subcutaneously in the ventral abdomen (Figure-15), with an offensive uremic odor emanating from the mouth. Results of USG examination included a collapsed bladder, uroperitoneum, and accumulation of urine in the testicles and ventral abdomen. On the basis of the clinical and USG findings, ruptured bladder and urethra were diagnosed, along with a grave prognosis. Unfortunately, no postmortem examination was done.

**Figure-16:**
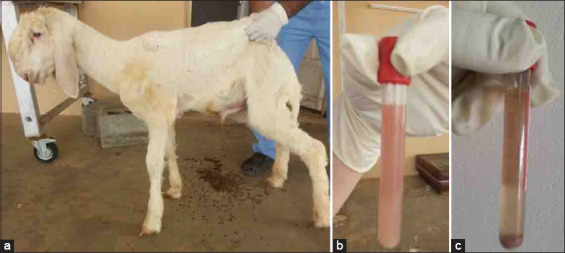
Paralysis of the urinary bladder in a lamb: (a) Incontinence with dribbling of urine as the main complaint; (b) cloudy urine sample; (c) urine sediment formed after centrifugation.

**Figure-17:**
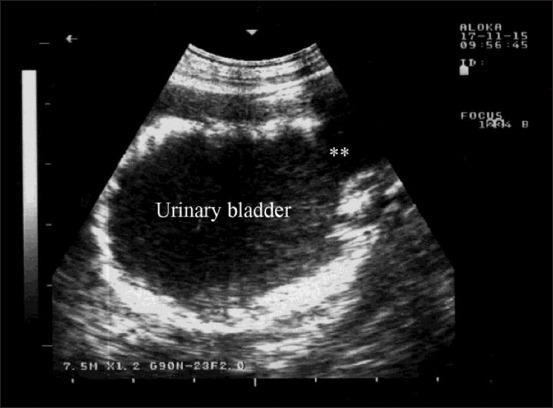
Ultrasonography of the urinary bladder in the lamb with paralysis of the urinary bladder as seen in Figure-16; thickened bladder wall and dilated bladder neck (stars) are evident. Image was taking using a 7.5 MHz convex transducer.

The lamb and goat in Group-10 with bladder paralysis presented with anorexia, dullness, and depression. Dribbling urine while walking and voiding small amounts of urine despite efforts at urination signaled urinary bladder dysfunction. Urine samples were cloudy. Clinical examination of the male lamb revealed an enlarged and edematous penis and testicles (Figure-16), with an offensive uremic odor emanating from the mouth. USG examination revealed a dilated bladder, thickened wall, hyperechoic sludge accumulating ventrally in the bladder, and a dilated bladder neck. Clear sediment formed after urine centrifugation (Figure-17).

## Discussion

To the author’s knowledge, this report describes the largest number of clinical, USG, and postmortem findings of different urinary pathologies in goats (n=28), as only two cases have been reported in the veterinary literature [17,18]. Simultaneously, 30 sheep with various urinary tract disorders were evaluated, presenting with pelvic abscessation, pigmented urine, renal failure, pyelonephritis, cystitis, hydronephrosis, ruptured urethra or urinary bladder, ruptured bladder with ruptured urethra, and bladder paralysis.

The pelvic abscesses in Group-1 were located close to the urinary bladder, compressing the bladder neck and causing bladder distension and urine retention. This consequently led to bilateral hydronephrosis, which was detected with USG and again on postmortem examination. Ulcerative cystitis, the direct cause of the deep-red urine in this group, could have been the sequelae of prolonged urine stasis. A similar case of urinary retention and ulcerative cystitis in a horse, as sequelae of pelvic abscessation, has been reported [20]. Hematuria and cystitis due to abdominal abscessation also have been reported in a 4.5-month-old foal [21], as were hematuria, hydronephrosis, aortic aneurysm, and ureteral obstruction secondary to umbilical artery abscessation in a 5-week-old foal [22].

The red urine presentation in Group-2 was a result of accidental grazing on a pasture containing toxic plants. Livestock can be poisoned by many plant species; however, an analytically confirmed diagnosis is not always easy, and most cases are suspected only following necropsy and the identification of leaves, stems, or pods in the rumen [23]. Plants that may be toxic to domestic animals include sorghum, greasewood, halogeton, water hemlock, Japanese yew, larkspur, lupine, milkweed, philodendron, oleander, castor bean, and precatory bean [24]. Unfortunately, the plants responsible for poisoning the animals in the current study were not determined. In sheep and goats, the most common cause of hemolytic anemia, hemoglobinemia, and hemoglobinuria is copper toxicosis [25,26].

In Group-3, the renal impairment manifested by marked elevations of BUN and creatinine may have been caused by neutrophilic leukocytosis.

Cases of pyelonephritis in sheep and goat have been reported as being caused by *Corynebacterium renale* [27,28]. Unfortunately, the causative agent of pyelonephritis in Group-4 in this study was not determined. Pyelonephritis is commonly diagnosed in cattle [29] and horses [30]. In cows, both transrectal and right flank USG has revealed dilatation of the right or left ureter, cystic lesions in one or both kidneys, and dilatation of the renal sinus. In the present study, kidney USG was carried out transcutaneously, revealing increased echogenicity of the renal parenchyma and bilateral dilatation of the renal pelvis and calices. Similarly, in a mixed-breed goat with cystitis and bilateral pyelonephritis, results of a USG examination revealed an increase in the magnitude of the right kidney and renal pelvis and calices, kidney enlargement, and a decrease in cortex and medulla separation [17].

The inflammation of the urinary bladder in Group-5 was consistently associated with frequent, painful urination; prolonged urination posture; thickened, corrugated bladder mucosa; and a decreased urine output. In farm animals, common associations with cystitis include cystic calculus, difficult parturition, contaminated catheterization, late pregnancy, and bladder paralysis, with *C. renale* as the predominant causative agent [31]. This disease occurs sporadically as a result of the introduction of infection into the bladder after trauma or when urine stagnation occurs. Diffuse thickening of the bladder wall is a common sonographic abnormality. The urethritis that typically accompanies cystitis causes painful sensations and frequent urination [31]. The current study’s USG results agree well with those of a goat with cystitis and bilateral pyelonephritis in which USG showed enlargement of the urinary bladder, urinary stasis, and bladder wall thickening [17].

The clinical presentation in Group-6 was nonspecific; loss of body condition was the only complaint reported by the owner. This issue is most commonly caused by urinary tract obstruction. Routine USG revealed dilated renal pelvis and calices. It is not always possible to scan the left kidney in sheep through the flank, but such an examination was not necessary because the urinary tract obstruction in males is distal to the ureter, and therefore, the condition affects both kidneys equally. These findings are consistent with previous reports of advanced hydronephrosis [32,33], in which the condition was identified by an increased renal pelvis, representing an enlarged anechoic (fluid-filled) center of the kidney, and a reduced renal cortex.

The largest group in this study, Group-9, had a ruptured urethra, which the clinical findings clearly indicated, as shown by enlarged and edematous penis and testicles. USG confirmed an intact bladder, subcutaneous and in-testicle urine accumulation, and urolithiasis within the penile body. Urethra rupture as a sequela of urolithiasis is common in small ruminants as compared to large ruminants, for which bladder rupture dominates [31]. Surgical tube cystotomy can be performed with an intact bladder [34,35].

In addition, anuria and abdominal distension were diagnosed in the group with a ruptured urinary bladder. USG confirmed a collapsed bladder and uroperitoneum. A similar case of obstructive urolithiasis in a Saanen goat resulting in a ruptured bladder has been reported [36]. Scott [32] has stated that rupture of the bladder resulting from obstructive urolithiasis is rare in rams, except for in cases of neglect [37], and that gross hydronephrosis develops over several days due to high urinary back pressure [32].

Rupture of both the urinary bladder and the urethra was seen in Group-9’s clinical presentations and USG findings. Urethral perforation or urinary bladder rupture has been reported in neglected cases of urethral obstruction [37]. Recently, diagnostic USG has been found to be useful in large animals to verify cases with obstructive urolithiasis causing either rupture of the bladder or the urethra [38,39].

In Group-10, the first diagnostic sign was dribbling urine while walking. Differential diagnosis may include urolithiasis and chronic cystitis; both were present in the lamb in this group with an enlarged and edematous penis and testicles. Primary urinary bladder paralysis and cystitis also have been reported in a goat with urolithiasis [33]. Bladder paralysis, which is uncommon in large animals, usually occurs as a result of neurological diseases affecting the lumbosacral spinal cord [31]. Dribbling and voiding small amounts of urine despite efforts at complete urination are major signs of bladder dysfunction, which was observed in this group. The hyperechoic sludge detected ventrally in the bladder indicated secondary cystitis.

## Conclusion

This study has two limitations. First, urinalysis and histopathological examinations were not performed. Second, the clinical presentations of many of the sheep and goats described in this study were nonspecific and in some cases directed the clinician to other diseases. Further research is therefore needed to correlate results of USG with histopathological findings in sheep and goats with urinary tract disorders. The current study found that USG was a superior tool for confirmatory diagnosis of urinary tract disorders; therefore, it is the preferred choice in veterinary practice.

## Author’s Contribution

MT: Designed and performed the experiment, analyzed the data, wrote and revised the manuscript, and prepared the tables. The author read and approved the final manuscript.
